# Development and comparison of a Chinese nomogram adding multi-parametric MRI information for predicting extracapsular extension of prostate cancer

**DOI:** 10.18632/oncotarget.11559

**Published:** 2016-08-23

**Authors:** Yuke Chen, Wei Yu, Yu Fan, Liqun Zhou, Yang Yang, Huihui Wang, Yuan Jiang, Xiaoying Wang, Shiliang Wu, Jie Jin

**Affiliations:** ^1^ Department of Urology, Peking University First Hospital, Xicheng, Beijing, China; ^2^ Institute of Urology, Peking University, National Urological Cancer Center, Beijing, China; ^3^ Department of Radiology, Peking University First Hospital, Xicheng, Beijing, China

**Keywords:** prostate cancer, radical prostatectomy, extracapsular extension, nomogram, multi-parametric magnetic resonance imaging

## Abstract

**Purpose:**

To improve the performation of a nomogram for predicting side-specific extracapsular extension (SS-ECE).

**Results:**

One hundred and ninety-six patients (55.5%) had ECE on final pathology. Bilateral and unilateral ECE rate was 13.9% (49/353) and 41.6% (147/353), respectively. The mean age was 65.9 years and the mean serum prostate specific antigen (PSA) was 15.0 ng/ml. Based on multivariate logistic regression analysis, clinical stage (cStage), PSA, Gleason sum, percentage of positive cores, and ECE risk score were significant predictors of ECE. The current nomogram had higher predictive accuracy (0.851) and superior calibration. According to the decision curve analysis (DCA) results, the updated nomogram demonstrated a high net benefit across a wide range of threshold probabilities.

**Materials and Methods:**

We studied 353 patients with cStage T1c-T3 prostate cancer underwent radical prostatectomy. The candidate predictors associated with ECE were cStage, PSA, Gleason sum, percentage of positive cores, maximum cancer percentage and ECE risk score from multi-parametric magnetic resonance imaging (MP-MRI). The receiver operating characteristic (ROC) analysis was performed and an updated nomogram was constructed. The DCA was performed to test the predictive ability of the nomogram. In addition, the validation and calibration of the Memorial Sloan-Kettering cancer center (MSKCC) nomograms were performed in the current subjects.

**Conclusions:**

Predictors, including cStage, PSA, Gleason sum, percentage of positive cores, maximum cancer percentage, and ECE risk score, were combined to construct a SS-ECE prediction nomogram. And the current nomogram might help urologists in decision-making process of preserving or resecting neurovascular bundles preoperatively.

## INTRODUCTION

The presence of extracapsular extension (ECE), which frequently occurs posterolaterally in the region of the neurovascular bundle (NVB), is not uncommon in patients with prostate cancer [1]. Resection of the NVB substantially decreases the chance of recovering erectile function, but preservation of the nerve may lead to a positive surgical margin. Therefore, it would benefit patients if surgeons documented the ECE as accurately as possible during the pre-operative evaluation.

To promote the accuracy of ECE prediction, numerous predictive models have been proposed. In 1997, Partin tables were constructed based on the prostate-specific antigen (PSA) level, Gleason score, and clinical stage. In 2001, Graefen et al. [2] enhanced the specificity of this approach by generating regression tree analysis capable of predicting the probability of ECE in a side-specific (SS) manner. This model facilitated the identification of candidates for non-, unilateral-, or bilateral nerve-sparing prostatectomy. Furthermore, the 2006 Memorial Sloan-Kettering cancer center (MSKCC) prostate cancer nomogram included supplemental information from the prostate biopsy [3].

Multi-parametric magnetic resonance imaging (MP-MRI) is increasingly accepted to be useful in detecting prostate cancer and ECE. Recently published clinical guidelines [4] from prostate MRI authorities have included a structured uniform reporting and scoring system (PI-RADS) and ECE risk score to standardize prostatic MRI readings. In 2015, Boesen et al. [5] verified the ECE risk score in predicting ECE with relatively high accuracy.

Herein we integrated information from the Partin tables and the MSKCC nomogram into the ECE risk score in MP-MRI to construct a new nomogram to predict the likelihood of ECE on each side of the prostate.

## RESULTS

Table 1 presented the descriptive statistics of the study cohort. The average age was 65.6 years and 66.3 years for non-ECE and ECE patients, respectively. The mean PSA was 12.9 ng/mL and 19.0 ng/mL for non-ECE and ECE patients, respectively (P<0.001). Histopathologic evaluation of the prostatectomy specimens revealed ECE in 196 of 353 patients (55.5%). Bilateral ECE was reported in 49 patients (13.9%), while unilateral left and right ECE was recorded in 87 (24.6%) and 60 (17.0%) patients, respectively. Lobe-specific ECE was present in 245 of 706 prostate lobes (34.7%). Based on univariate analyses, all variables demonstrated statistical significance except for age (p=0.220). Based on multivariate logistic regression analyses, cStage (p <0.001), PSA (p=0.004), Gleason sum (p =0.035), the percentage of positive cores (p=0.009), and ECE risk score (p<0.001) were significant predictors of ECE unlike the maximum cancer percentage (Table 1).

**Table 1:** Factors that predict side-specific ECE based on univariate and multivariate analyses

Variable	ECE		Univariate	Multivariate	
	- (*n*= 461)	+ (*n*= 245)	*P*	OR (95%CI)	*P*
Age (years)	65.6±7.0	66.3±6.5	0.220	-	-
cStage (%)					<0.001
T1c	335 (80.5)	81 (19.5)		ref	-
T2a	103 (52.6)	93 (47.4)		1.85(1.17-2.93)	0.009
T2b	11 (19.3)	46 (80.7)		12.04(5.48-26.42)	<0.001
T2c	10 (33.3)	20 (66.7)		7.71(3.20-18.76)	<0.001
T3	2 (28.6)	5 (71.4)		5.72(0.92-35.60)	0.062
PSA (ng/ml)	12.9±9.7	19.0±14.8	<0.001	1.03(1.01-1.04)	0.004
Gleason sum(%)					0.035
≤6	139 (78.1)	39 (21.9)		ref	-
3+4	173 (70.9)	71 (29.1)		0.86(0.49-1.51)	0.601
4+3	71 (54.6)	59 (45.4)		1.94(1.04-3.61)	0.038
≥8	78 (50.6)	76 (49.4)	<0.001	1.31(0.70-2.45)	0.397
% Pos cores*	16.7[0.0, 40.0]	60[20, 83.^3^]	<0.001	1.01(1.00-1.02)	0.009
Max Ca %*	10.0[0.0-38.5]	71[28,71]	<0.001	1.01(1.00-1.02)	0.138
ECE risk score(%)					<0.001
0	140(85.9)	23(14.1)		ref	-
1	237(75.0)	79(25.0)		0.92(0.50-1.67)	0.778
3	63 (45.3)	76 (54.7)		3.49(1.83-6.66)	<0.001
4	16 (25.8)	46 (74.2)		4.24(1.80-9.97)	0.001
5	5 (19.2)	21 (80.8)	<0.001	7.25(2.23-23.63)	0.001

^*^% Pos cores (percent of positive cores) and Max Ca % (maxium cancer percent) were prensented as medians (q1, q3).

T test was used to compare age and PSA; Willcoxon test was used to compare cStage, Gleason score, % Pos cores and Max Ca % and ECE risk score.

Forward stepwise method was used for variable selection in binary logistic regression.

To assess the accuracy of predicting ECE, we calculated the area under the ROC curve (AUC) for each variable, the current three models and the MSKCC base and full models [3] in Table 2. The highest and lowest values of the AUC for single variables were 0.738 (ECE risk score) and 0.631(Gleason sum), respectively. For the three combined models, the AUC values were 0.792, 0.823, and 0.851 for the first, second, and third models, respectively, which were higher than any individual predictor variable alone. And the comparisons of AUC values between any of the two models were significantly different. In addition, the AUC values of the MSKCC base and full models were 0.770 and 0.796, which were significantly inferior to that of the corresponding first and second models in the current study (P=0.021 and 0.003).

**Table 2:** Predictive accuracy for SS-ECE based on ROC

	AUC (95% CI)
Individual predictive features:	
cStage	0.720(0.679-0.761)
PSA	0.658(0.616-0.700)
Gleason sum	0.631(0.588-0.674)
% Pos cores*	0.736(0.698-0.775)
Max Ca%*	0.724(0.685-0.763)
ECE risk score	0.738(0.698-0.777)
Combined predictive features:**	
cStage + PSA + Gleason sum	0.792(0.757-0.827)
cStage + PSA + Gleason sum+ % Pos cores + Max Ca%	0.823(0.791-0.855)
cStage + PSA + Gleason sum+ % Pos cores + Max Ca% + ECE risk score	0.851(0.822-0.881)
MSKCC base model [3]	0.770 (0.734-0.806)
MSKCC full model [3]	0.796 (0.763-0.830)

p(AUC[model1]vs.AUC[model2])=0.006; p(AUC[model2]vs.AUC[model3])=0.001; p(AUC[model1 vs. MSKCC base model])=0.021; p(AUC[model2 vs. MSKCC full model])=0.003.

*% Pos cores:percentage of positive cores; Max Ca%: maximum cancer percentage

**AUC values of first, second, and third models

**Figure 1:**
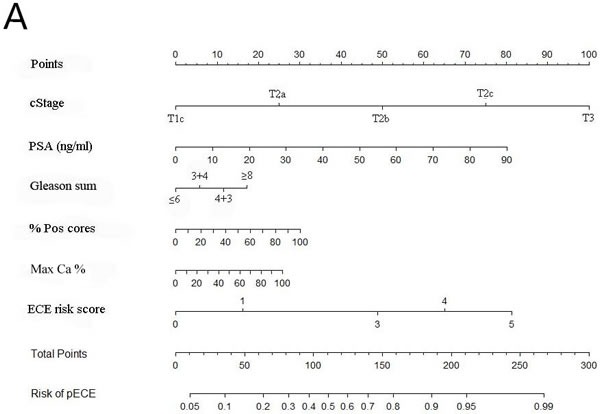

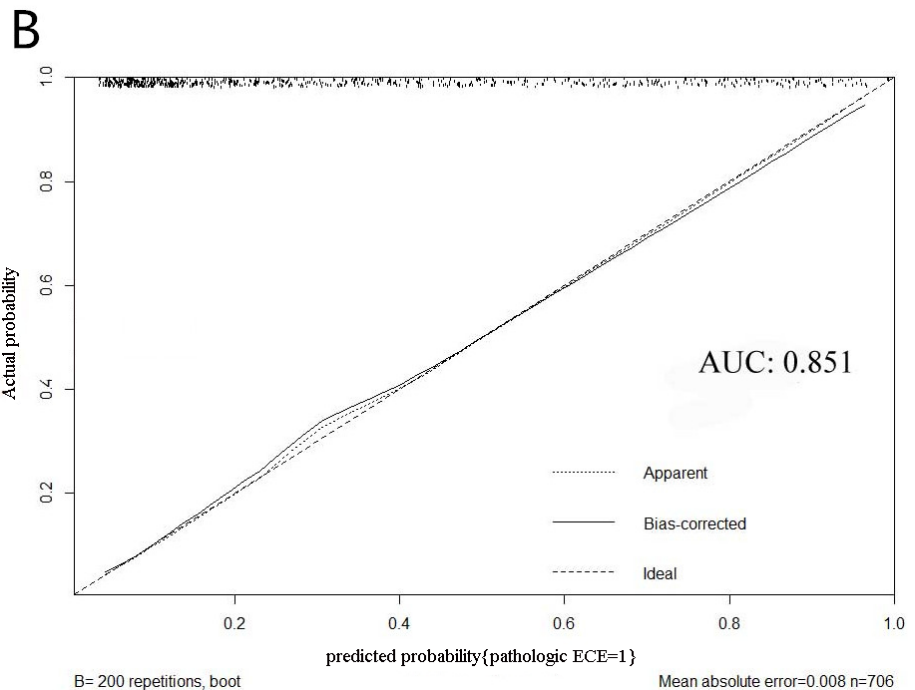
**A**. The updated nomogram predicting SS probability of ECE; **B**. Calibration of the nomogram (200 bootstrap re-samples).

The updated nomogram for predicting ECE was constructed based on the logistic regression (Figure 1A). The nomogram included cStage, PSA, Gleason sum, the percentage of positive cores, the maximum cancer percentage, and ECE risk score. The nomogram was used by first locating the patient position on each predictor variable scale. Each scale position had corresponding prognostic points (top axis). The points for each variable were added and the probability of ECE was estimated from the bottom line. For example, if there was a clinical stage T2b nodule on 1 side, the serum PSA was 20 ng/ml, the biopsy Gleason sum was 7 (4+3), the percentage of positive cores 50%, the maximum cancer percentage was 40%, and the ECE risk score was 3, the probability of ECE by the third model would be 70% for this single lobe. Figure 1B presented the calibration (200 bootstrap re-samples) of the nomogram related to actual outcomes for the 706 prostate lobes. The x-axis was the prediction calculated with the nomogram and the y-axis was the actual presence of ECE in each lobe of the prostate. The dotted line represented the performance of an ideal nomogram in which the predicted outcome perfectly corresponded to the actual outcome. The performance of the nomogram was plotted in 2 ways. The dashed line was the apparent accuracy without correction for overfit. The solid line was the bootstrap corrected nomogram performance, a scatter estimate of future accuracy. Note that because the solid line was close to the dotted line, the predictions calculated by the nomogram corresponded accurately with the actual outcomes.

**Figure 2:**
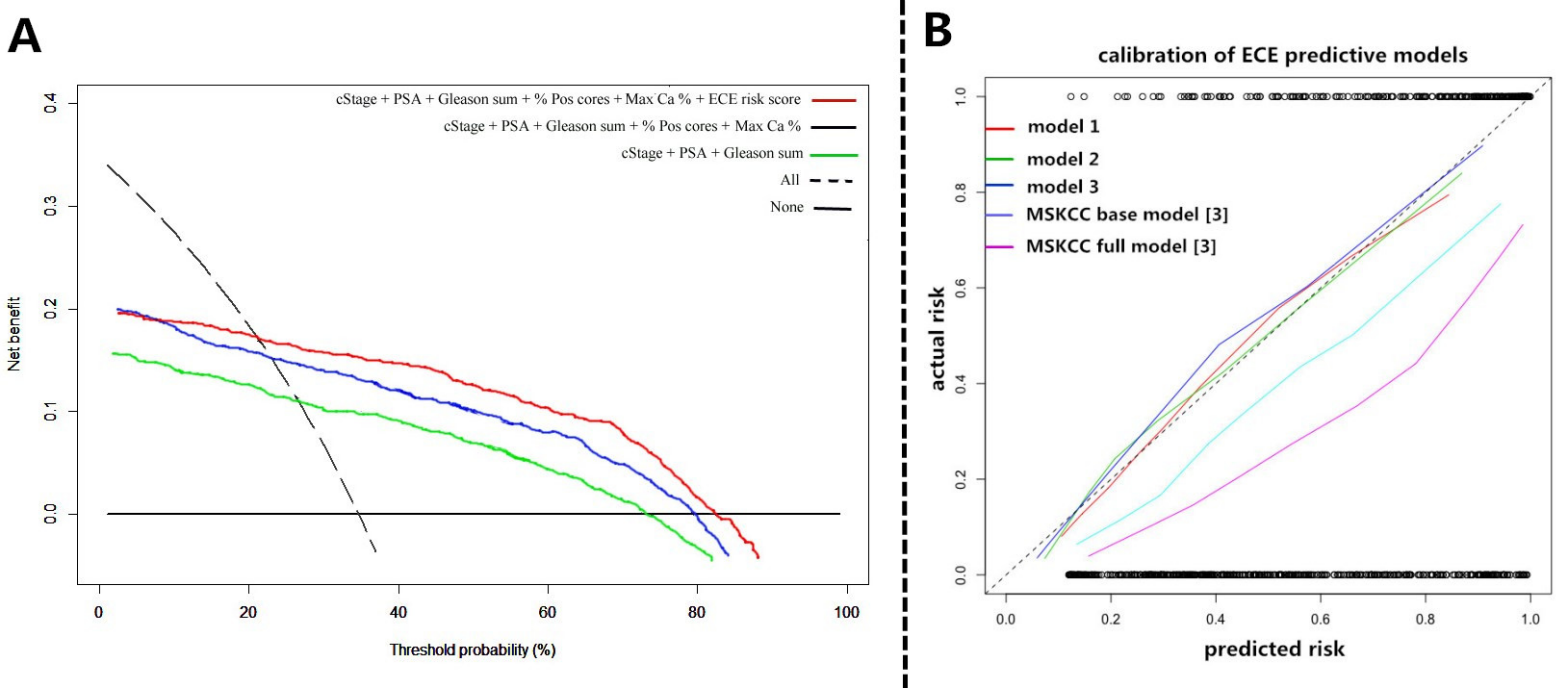
**A**. Decision curves for the three prediction models in the whole cohort. The y-axis measures net benefit, calculated by summing the benefits (true positives) and subtracting the harms (false positives), in which the latter are weighted by a factor related to the relative harm of a neglected ECE compared with the harm of missed diagnosis of ECE. A model is of clinical value if it has the highest net benefit compared with single predictors or other models. Decision analysis demonstrated a high net benefit across a wide range of threshold probabilities for the third model (red line); **B**. The three models of the current study were compared by MSKCC base and full models [3] for prediction of ECE by decimal of predicted risk. Better calibration was observed for the new developed models compared with MSKCC base and full models.

In Figure 2A, the results from the decision curve analysis (DCA) of SS-ECE predictability for the three models were presented. The first and third models had the lowest and highest net benefit across the entire spectrum of probability thresholds, respectively. The updated nomogram (third model) added value to a range of threshold probabilities between 22% and 83%, suggesting a benefit in patients with a probability within this range.

When it comes to the calibration part, the three models of the current study were compared by the MSKCC base and full models [3] for prediction of ECE by decimal of predicted risk (Figure 2B). Better calibration was observed for the new developed models compared with the MSKCC base and full models. And the risk of ECE was over estimated in all probabilities for the two MSKCC models.

In addition, the surgical pathologic characteristics of the 353 subjects were presented in Supplementary Table 1.

## DISCUSSION

As the prostate cancer is thought to differ epidemiologically and biologically between Western and Chinese populations, the performation of nomograms developed for other populations was not ideal for the Chinese prostate cancer population according to the published studies [6] and the validation and calibration results of the MSKCC nomograms among the subjects of the current study (Table 2 and Fgure 2B) [3]. Therefore, we developed a new predictive nomogram based on the data of prostate cancer in Chinese population, adding the ECE risk score based on MP-MRI [4, 5]. Not only would the new developed nomogram be used for preoperative prediction for Chinese PCa patients, but also some non-invasive treatment, included brachytherapy, high-intensity focused ultrasound (HIFU), cryosurgical ablation of the prostate (CSAP), etc, could be performed for low ECE probability patients based on individual operation tolerance assessment [7].

The reported incidence of ECE in prostate cancer varied widely in different studies. In the study conducted by Steuber et al. [3], the ECE rate was 303 of 1,118 (27%) among the American patients, which included 82 bilateral ECE patients (7.3%) and 221 unilateral ECE patients. Twenty-six of 112 prostate cancer patients had ECE in the Feng et al. [8] study conducted in the US in 2015. In 2010, Satake et al. [9] from Japan reported that the ECE rate was as high as 41.1% (146 of 354 patients). The ECE rate was 55.5% (196 of 353 patients). The discrepancy between the aforementioned studies might be attributed to the following reasons. First, approximately 75% of the patients (264 of 353) had a Gleason sum ≥7, which was higher than the 34% and 58% in the Steuber et al. [3] and Feng et al. [9] studies. And high grade of gleason score had been proven to be one of independent risk factors for ECE [1-3, 8, 9]. Second, it had been reprorted that differences of lifestyle and genetic factor (Single nucleotide polymorphisms, SNPs), low sensitivity of the PSA test, delayed healthcare, etc. would also act [10-13]. In 2014, Chen et al. [13] reported that the rates of high-grade (Gleanson sum≥7) and organ extension (cStage≥T3) were more than 70% and 55% at initial diagnosis among prostate cancer patients from forty-one medical centers all over China, which were consistent with the current study. In addition, the occurrence rate of seminal vesical invasion (SVI) was similar with the results from some other Asian studies [14] (16.4% vs. 17%) and relative higher than that of some Western reports [15] (Supplementary Table 1). Therefore, performation of predictive model for side specific ECE in combine with considering probabilities of SVI and lymph node metastasis would utmost preserve the erectile function, especially for prostate cancer cohorts of high grade risk.

Nomograms are widely used for cancer prognosis, primarily because nomograms reduce statistical predictive models into a single numerical estimate tailored to the profile of an individual patient. User-friendly graphical interfaces for generating these estimates facilitate using nomograms to inform clinical decision making [16]. Ohori et al. [1] from the USA and Steuber et al. [3] from Europe generated the famous MSKCC nomograms based on cStage, PSA, Gleason sum, percentage of positive cores, and percentage of cancer, thus providing predictive accuracies for SS-ECE of 0.806 and 0.840, respectively. In 2010, Satake et al. [9] from Japan generated a nomogram with the same clinical data acquiring an AUC value of 0.797. Similarly, the predictive accuracy of our second model was 0.823.

Recent findings support the rapidly growing use of MP-MRI as an efficient imaging tool for prostate cancer staging [17]. Gupta et al. [18] reported that compared with the performance of the newest iteration of the Partin tables, MP-MRI is better for tumor staging. In 2015, Boesen et al. [5] evaluated the diagnostic performance of pre-operative MP-MRI with an ECE risk score in prostate cancer tumor stage and prediction of ECE at final pathology. When the cut-off level was set at ≥ 4, the ROC curve analysis of the ECE risk score revealed a high AUC. In the current study, when the ECE risk score was used without a cut-off value, the predictive value for this single variable was 0.738, which was > 0.701 at the cut-off value ≥4. Also, it had been demonstrated that multivariate models, such as nomograms, are more accurate if variables were used in the original format without transformation [3, 16]. Therefore, we updated the nomogram to add the ECE risk score in MP-MRI without a cut-off value, significantly enhancing the predictive accuracy from 0.823 to 0.851 (P=0.001).

Feng et al. [8] added the PI-RADS to the MSKCC nomogram to boost the predictive accuracy as high as 0.94; however, the predictive accuracy of the MSKCC nomogram in the Feng et al. study [8] was 0.86, which was also relatively high among the existing study [1, 3, 9]. In addition, all of the patients in the Feng et al. study [8] underwent imaging using a 3.0 T MRI system without an endorectal coil. In contrast, 32% (113 of 353) and 68% (240 of 353) the patients underwent 1.5T and 3.0 T MP-MRI without an endorectal coil, respectively. According to Nardo et al. [19] and Shah et al. [20], 3.0 T MRI was superior at image quality, fat suppression, and amount of artifacts. Yet, considering the diagnostic performance, visualization of anatomic structures, and clinical abnormalities, there were no significant differences between the two types. Even in developed countries, like the US, the usage rate of 3.0 T MRI was only 59% in non-academic clinical practices [21]. Meanwhile, the usage of an endorectal coil in 1.5 T MRI is still a matter of contention [4, 20-24]. Lee et al. [22] and Kim et al. [23] suggested that the use of endorectal coil MRI did not significantly improve the staging of prostate cancer and presented several complications in terms of diagnostic accuracy and patient comfort. Therefore, data from 1.5 T MRI without an endorectal coil were not excluded [24].

There were several limitations to our study. Our predictive nomogram for detection of ECE was calibrated with 200 bootstrap resamples, which would result in overfitting. Therefore, future studies are needed to validate our model with other data sets. Selection bias is inevitable for all the retrospective studies. MP-MRI might more likely be performed depending on the experience of the urologist without a uniform standard. Instead of using SS Gleason sum, the total Gleason sum of one patient was integrated into our nomogram, which might cause a relatively low predictive value of this single variable. We found this more reflective of everyday clinical practice.

## MATERIALS AND METHODS

### Subjects

Between January 2009 and December 2015, we performed a detailed, systematic assessment of pre-operative clinical and post-operative pathologic findings in 1049 patients with integral prostate specimens. Further, 903 prostate cancer specimens were left after excluding the confounding factors from a part of radical cystoprostatectomy specimen of bladder cancer paients (n=146). These 903 patients had biopsy-proven surgically correctable prostate cancer, and then underwent radical prostatectomy (RP) with or without staging lymphadenectomy at our institution (Peking University First Hospital). The following clinical information were evaluated: age; pre-treatment PSA; results of SS digital rectal exam (DRE); SS data from TRUS-guided prostate systematic needle biopsies; SS pathologic findings; and SS MP-MRI findings. The exclusion criteria included patients receiving neoadjuvant hormornal therapy (NHT) or radiation therapy, having prostate biopsy in other medical centers, with contraindications to MP-MRI, and lack of detailed clinical information.

Overall, 550 patients who received neoadjuvant therapy (n=145) or had incomplete clinical information or other hospitals biopsy results (n=405) were excluded. Three hundred fifty-three patients met our inclusion criteria, thus the patient data were used for further analysis. All of the subjects were informed about the nature and intended purpose of the study. A document of informed consent, which was approved by the Ethics Committee, was signed by all of the subjects. This study was approved by the Ethics Committee of Peking University First Hospital.

### Clinical stage

Clinical stage was assigned based on the 2002 TNM staging system to each side of the prostate. For example, a patient with a T2a cancer would be considered to have T1c cancer on the side with no palpable abnormalities, while a patient with palpable cancer on both sides was considered to have a T2c cancer on each side [1-3].

### Pathologic evaluation

By two experienced doctors, transrectal ultrasound- (TRUS-) guided systematic biopsies of ≥10 cores (usually 12 or 13 needles) were performed in all patients. All biopsy and surgical specimens were evaluated by dedicated genitourinary pathologists. For each core of the biopsy specimens, we recorded the location, primary and secondary Gleason scores, the length of cancer (mm), and the length of each core. On each prostate lobe, we calculated the percentage of positive cores, and the maximum cancer percentage was defined as the greatest tumor percentage among the positive cores of biopsies. The surguical prostate specimens were subjected to overnight fixation in 4% buffered formalin, then being inked and totally embedded. Specimens were sliced into several segments (usually quadrants). The apex and two lobes of a prostate specimen were examined separately. The seminal vesicles were cut longitudinally and totally embedded. The ECE was defined as invasion of the prostate cancer beyond the prostate capsule into the peri-prostatic soft tissues on post-operative pathologic evaluation. If tumour extended beyond the confines of the gland on only one lobe of the prostate, ECE was recorded as unilateral; whereas if it was present on both left and right sides, it was designated as bilateral [25].

### MP-MRI

The majority of MP-MRI information was obtained before biopsy or 4 weeks after biopsy to reduce the influence of hemorrhage. All patients underwent MP-MRI using a 1.5T (113 of 353 patients) or 3.0 T (240 of 353 patients) MRI scanner without an endorectal coil. Biplanar T2W images from below the prostatic apex to above the seminal vesicles were obtained. In addition, axial diffusion-weighted images (DWIs), including three b-values (b0, b800, and b1000), along with reconstruction of the corresponding apparent diffusion coefficient (ADC) map (b-values 800 and 1000), and dynamic contrast-enhanced (DCE) images before, during, and after intravenous administration of contrast agent were performed. The ECE tumor characteristics were assessed according to the following findings: a) score 0, no sign of ECE; b) ECE risk score 1, capsular abutment; c) ECE risk score 3, capsular irregularity, retraction, or thickening; d) ECE risk score 4, neurovascular bundle thickening and capsular signal loss or bulging; and f) ECE risk score 5, direct sign of tumor tissue in the extraprostatic tissues [4, 5]. Two experienced radiologists retrospectively and independently interpreted the MRI images. Any disagreement in interpretation of the results was resolved by the senior radiologist.

### Statistical analysis

SPSS version 20.0 (IBM Corporation, USA) and R version 3.1.3 (R foundation for Statistical Computing, Vienna, Austria) were used for statistical analysis. Categorical variables were presented as frequencies and percentages, and continuous variables were presented as means and standard deviations (SDs) or medians (q1 and q3). The T test was used to compare the age and PSA; The Willcoxon test was used to compare the cStage, Gleason score, % Pos cores and Max Ca % and ECE risk score. Forward stepwise method was used for variable selection in binary logistic regression. We constructed three predictive models. The first model consisted of the three predictors in Partin tables (PSA, cStage, and Gleason sum). The second model added the percentage of positive cores and the maximum cancer percentage on each side to the first model, which was consistent with the MSKCC prediction model [3]. The third model added the ECE risk score on each side to the second model. The predictive accuracy was determined for each variable and each model, which were quantified with receiver operating characteristic (ROC) curve analysis. In addition, the MSKCC base and full models were validated by the data of the current study in the form of ROC curve analysis. Area under the curve (AUC) was calculated for each model and compared against each other by the z-test. A P<0.05 was considered to be statistically significant. Furthermore, calibration curves assessed the agreement between the actual ECE risk and the predicted probabilities of the three models in the current study and the MSKCC base and full models with an intercept (ideally to be 0) and as lope (ideally to be 1). The nomogram was developed based on the logistic regression for the third model, using 200 bootstrap re-samples to decrease the overfit bias. Decision curve analysis (DCA) was performed to compare the accuracy of the three models by calculating the net benefit over a spectrum of probability thresholds [26].

## CONCLUSIONS

We constructed a nomogram for predicting SS-ECE based on the results of cStage, PSA, Gleason sum, percentage of positive cores, maximum cancer percentage, and ECE risk score in Chinese patients. The nomogram provides an accurate prediction of ECE and adds value to a wide range of threshold probabilities. And the current nomogram might help urologists in decision-making process of preserving or resecting neurovascular bundles preoperatively.

## SUPPLEMENTARY MATERIALS FIGURES AND TABLES


