# Supplementary material for: Development and comparison of a Chinese nomogram adding multi-parametric MRI information for predicting extracapsular extension of prostate cancer
Source: Oncotarget. 2016 Aug 23;8(13):22095–103. doi: 10.18632/oncotarget.11559 (PMC5400649; doi:10.18632/oncotarget.11559)
Supplement: Supplementary file 1 [file oncotarget-08-22095-s001.pdf]

Development and comparison of a Chinese nomogram adding Multi-parametric MRI information for predicting extracapsular extension of prostate cancer

Supplementary Material

Supplementary Table 1: Pathologic characteristics of the 353 subjects

Pathologic outcomes	N(%)
Gleason score	
≤ 6	30 (8.5)
3+4	135 (38.2)
4+3	107 (30.3)
≥ 8	81 (23.0)
seminal vesicle invasion	
positive	58 (16.4)
negative	295 (83.6)
surgical margin	
positive	101 (28.6)
negative	252 (71.4)
extracapsular extension	

positive	196 (55.5)
negative	157 (44.5)
lymph node metastasis	
positive	8 (2.3)
negative	203 (57.5)
without lymphadenectomy	142 (40.2)
